# Anatomical Variant of Spinal Accessory Nerve Passing through Fenestrated Internal Jugular Vein

**DOI:** 10.1155/2022/7087970

**Published:** 2022-03-26

**Authors:** Shohei Takaoka, Kenji Yamagata, Makiko Okubo-Sato, Satoshi Fukuzawa, Fumihiko Uchida, Naomi Ishibashi-Kanno, Hiroki Bukawa

**Affiliations:** ^1^Department of Oral and Maxillofacial Surgery, University of Tsukuba Hospital, Tsukuba, Ibaraki 305-8576, Japan; ^2^Department of Oral and Maxillofacial Surgery, Institute of Clinical Medicine, Faculty of Medicine, University of Tsukuba, Tsukuba, Ibaraki 305-8575, Japan

## Abstract

Neck dissection (ND) is a major surgery for head and neck cancer. Currently, some or all of the spinal accessory nerve (SAN), sternocleidomastoid muscle, and internal jugular vein (IJV) are aggressively preserved during ND to reduce postoperative complications. Since the anatomical relationship between the SAN and IJV has several variations, knowledge of these variations is necessary to avoid iatrogenic damage. In the present case, the SAN was observed to pass through the fenestrated IJV at the level of the posterior belly of the digastric muscle during ND in a patient with squamous cell carcinoma of the mandible. Although the anatomical structure of the SAN and IJV is rare, surgeons must be aware of this anatomical variation.

## 1. Introduction

Neck dissection (ND) is the principal technique for the treatment, prevention, and diagnosis of cervical lymph node metastasis in head and neck cancer. In the early 1900s, radical neck dissection (RND) was reported to remove all lymph nodes belonging to levels I to V, as well as the spinal accessory nerve (SAN), sternocleidomastoid muscle (SCM), internal jugular vein (IJV), and other surrounding tissues [1]. However, this surgery is highly invasive and results in a significant functional loss. In order to prevent postoperative deformity and loss of function, in the 1960s, the concept of functional ND was introduced [2, 3]. The aim of this surgery is to aggressively preserve the SAN, SCM, and IJV. Currently, modified radical neck dissection (mRND) is widely performed based on the principle of functional ND [4]. Mills et al. reported four anatomical relationships between the SAN and IJV [5]. Although the most common variant is the SAN passing laterally to the IJV, the SAN rarely passes through the fenestrated IJV in 0.9%-2.8% of cases [6–10]. Severing or damaging the SAN during ND results in motor deficits such as neck rotation and upper arm elevation. To increase awareness and reduce morbidity associated with iatrogenic SAN injury, surgeons must be aware of these anatomical variations and anticipate them when following the course of the SAN.

In this report, we present a rare case of the SAN passing through the window between the branches of the IJV and also review previous reports regarding this anomaly.

## 2. Case Presentation

**Figure 1:**
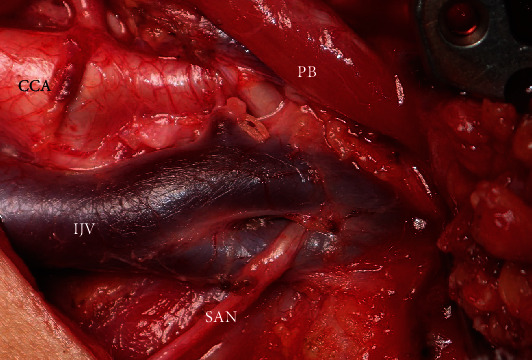
Intraoperative photograph, SAN passes through fenestrated IJV. SAN: spinal accessory nerve; IJV: internal jugular vein; CCA: common carotid artery; PBDM: posterior belly of the digastric muscle.

A 62-year-old woman presented with a T4bN2bMo squamous cell carcinoma of the left mandibular gingiva. mRND was performed prior to segmental mandibulectomy and mandible reconstruction with a free fibula flap, using the facial artery and IJV as recipient vessels. At the level of the posterior belly of the digastric muscle (DM), the IJV branched off, with the subsequent rejoining of the branched blood vessels. Fenestration of the IJV was encountered approximately 10 mm above the bifurcation of the common carotid artery. The SAN passed through the window of the IJV and coursed medially to the anterior part and laterally to the posterior part of the window (Figure 1). The operative time was 11 h and 20 mins, and the bleeding volume was 277 mL. The postoperative course was uneventful, and there was no functional disorder of the shoulder. The patient is alive with no primary or regional recurrence as confirmed at the two-year postoperative follow-up.

## 3. Discussion

ND was first performed approximately 100 years ago, and preservation of the SAN, SCM, and IJV has become a basic concept to reduce postoperative complications. Therefore, it is imperative for surgeons to possess knowledge of their anatomical variations to reduce the risk of injury. Mills et al. reported four anatomical relationships of the SAN and IJV at the level of the posterior belly of the DM [5]. The SAN crosses (1) laterally, (2) medially to the IJV, (3) passes through the fenestrated IJV, or (4) goes around the IJV. The most common anatomical variant of the SAN and IJV is the SAN passing laterally to the IJV and is observed in 71.7%-96.6% cases [6–8, 11], while SAN is reported to pass medially to the IJV in 2.6%-28.3% cases [6–8, 11]. The incidence of SAN passing through the fenestrated IJV is 0.9%-2.8% [6–10]. Furthermore, Johal et al. reported that the SAN divided and coursed both laterally and medially around the IJV in 0.5% cases [12].

The SAN has two origins, the brainstem and the spinal cord. The brainstem-derived nerve innervates the pharyngeal and other muscles along with the vagus nerve, and the spinal roots of the SAN innervate the SCM and trapezius muscles [12]. The SAN passes through the jugular foramen and crosses lateral or medial to the IJV. SAN is the commonly used landmark for ND. The posterior belly of the DM was encountered after the resection of the inferior border of the parotid gland. The IJV and SAN were observed at the level of the posterior belly of the DM.

The reason for the passage of SAN through the fenestrated IJV is still unclear. The vascular embryological theory, which is the most acceptable theory for this variation, states that the fenestrated IJV may be caused by inadequate condensation of the embryonic capillary plexus [13]. If part of the venous ring remains at the time of IJV formation, the formation of a window of the IJV around these veins could occur, and this anatomical structure is completed when the SAN enters the window [13]. Few study reports have identified the IJV fenestration; however, the SAN crossed the central part of the IJV and did not pass through the window [14, 15]. Moreover, these cases cannot be explained by vascular embryological theory alone. Thus, further embryological research on both SAN and IJV is needed.

Preoperative diagnosis is crucial for identifying structures with anatomical variations. Abakay et al. reported the importance of ultrasonographic investigation, as they were able to detect fenestration of the IJV using ultrasonography [16]. Hashimoto et al. and Towbin et al. reported that IJV fenestrations were able to observe on contrast-enhanced CT imaging [10, 17]. However, Hashimoto et al. reported that it is very difficult to detect small-fenestration IJV with CT or MRI [10]. To prevent surgical complications, further trials on preoperative examinations to understand the anatomical structures of the SAN and IJV are needed.

## 4. Conclusion

SAN may penetrate the fenestrated IJV at the level of the posterior belly of the DM. Surgeons must be made aware of this rare anatomical anomaly, perform preoperative diagnosis, and identify the SAN and IJV with attention to its anatomical variation during ND.

## Data Availability

The data used to support the findings of this study are included within the article.
